# One scale to rule them all: interpretable multi-scale Deep Learning for predicting cell survival after proton and carbon ion irradiation

**DOI:** 10.3389/frai.2026.1807677

**Published:** 2026-06-26

**Authors:** Giulio Bordieri, Giorgio Cartechini, Anna Bianchi, Anna Selva, Valeria Conte, Marta Missiaggia, Francesco G. Cordoni

**Affiliations:** 1 Department of Physics, University of Trento, Trento, Italy; 2 Trento Institute for Fundamental Physics and Application (TIFPA), Trento, Italy; 3 Department of Radiation Oncology (Maastro), GROW School for Oncology and Reproduction, Maastricht University Medical Centre, Maastricht, Netherlands; 4 Istituto Nazionale di Fisica Nucleare – Laboratori Nazionali di Legnaro, Legnaro, Italy; 5 Department of Physics and Astronomy, Louisiana State University, Baton Rouge, LA, United States; 6 Department of Civil, Environmental and Mechanical Engineering, University of Trento, Trento, Italy

**Keywords:** Deep Learning, interpretability, particle therapy, predictive modeling, radiation quality, radiobiology

## Abstract

**Introduction:**

The relationship between the physical characteristics of the radiation field and biological damage is central to both radiotherapy and radioprotection, yet the link between spatial scales of energy deposition and biological effects remains not entirely understood.

**Methods:**

To address this, we developed an interpretable Deep Learning (DL) model that predicts cell survival after proton and carbon ion irradiation, leveraging sequential attention to highlight relevant features and provide insight into the contributions of different energy deposition scales. Trained and tested on the Particle Irradiation Data Ensemble (PIDE) dataset, our model incorporates, in addition to linear energy transfer (LET), nanodosimetric and microdosimetric quantities simulated with MC-Startrack and Open-TOPAS, enabling multi-scale characterization.

**Results and discussion:**

While achieving high predictive accuracy, our approach also emphasizes transparency in decision-making. We demonstrate high accuracy in predicting relative biological effectiveness (RBE) for *in vitro* experiments. Multiple scales are used concurrently, with no single spatial scale predominating. Quantities defined at smaller spatial domains generally have a greater influence, whereas the LET plays a lesser role.

## 1 Introduction

Ionizing radiation therapy remains a cornerstone in the treatment of approximately half of patients with localized malignant tumors (Baskar et al., 2012; Durante and Flanz, 2019). Among the available modalities, particle therapy, particularly with protons and carbon ions, has gained clinical adoption due to its superior dose distribution and enhanced tissue sparing compared to conventional X-rays (Durante and Loeffler, 2010). By the end of 2023, more than 410,000 patients worldwide had received particle therapy, with nearly 350,000 treated using protons (PTCOG, 2024).

As particle therapy continues to expand, a deeper understanding of the biological effects of radiation is essential for improving therapeutic efficacy while minimizing adverse outcomes. Radiobiological models must therefore account for both the inherently stochastic nature of radiation-matter interactions and the complex downstream biological response mechanisms.

Recent research has increasingly focused on optimizing physical parameters to influence biological outcomes, with particular emphasis on linear energy transfer (LET) optimization, microdosimetric approaches, and nanodosimetric approaches (Romano et al., 2014; Faddegon et al., 2023; Cartechini et al., 2024). These strategies differ primarily in the spatial scale at which energy deposition is considered. LET optimization addresses average energy deposition along particle tracks at a macroscopic level. Microdosimetry examines energy deposition patterns at the micrometer scale, relevant to cellular structures. Nanodosimetry focuses on the nanometer scale, where individual ionization clusters within DNA-sized volumes are analyzed.

However, these strategies often either rely on non-stochastic descriptors such as LET or account for stochastic energy deposition only at a single characteristic domain size, thereby neglecting the multiscale complexity of radiation interactions. One open question remains as to whether a specific domain size exists that is most relevant for predicting the biological effect of radiation and, consequently, treatment efficacy. In fact, importantly, it is believed these three approaches are complementary, as different spatial scales are associated with distinct biological endpoints: macroscopic approaches often relate to tumor control and normal tissue complication probabilities, microdosimetric analyses inform cell survival and repair processes, while nanodosimetric studies are linked to complex DNA damage and mutagenesis (Missiaggia, 2024). In this context, the introduction of both nano- and micro-scale perspectives alongside the commonly used macroscopic LET appears essential. Different scales likely provide complementary information, as each reflects energy deposition in distinct biological targets (Friedrich et al., 2018). While it is widely acknowledged that radiation effects manifest across multiple spatial scales, as in demonstrated by the many radiobiological multiscale models (Surdutovich and Solov'yov, 2014; Cunha et al., 2017; Bordieri et al., 2025), there is currently no consensus on which of these provides the most accurate or mechanistically informative link to cellular survival outcomes (Missiaggia, 2024).

This work provides a detailed data-driven investigation of radiation quality across multiple spatial domains by integrating nanodosimetric and microdosimetric information alongside conventional dosimetric variables. It addresses the complex challenge of understanding how energy deposition, described at different levels, affects the overall biological impact of radiation on cell survival. In this context, Deep Learning (DL) methods allow us to identify patterns and make predictions directly from data. DL relies on artificial neural networks composed of multiple layers that can automatically extract and combine features from large datasets to capture non-linear dependencies and complex feature interactions. Recent applications of neural network-based approaches in this context include (Sciuto et al., 2025), which employs a neural network to estimate early DNA damage across micro- and nanometric scales.

Despite DL's high predictive accuracy, a major challenge lies in its interpretability. To address this issue, we develop a DL model to predict the biological effects of radiation on tissues, achieving both high predictive accuracy and built-in interpretability through an attention mechanism. We leverage sequential attention to dynamically select relevant features at each decision step, enabling native interpretability and efficient learning through sparse attention, forcing the model to focus on specific features at each step. This approach allows us to gain insights into the contributions of different energy deposition scales to biological damage while maintaining transparency in the model's decision-making process.

A DL model for cell survival prediction is trained to learn patterns from radiobiological and physical features and evaluated on independent datasets to assess its ability to generalize and provide biologically interpretable insights into post-irradiation outcomes. In this work, we focus on proton and carbon-ion cell-survival experiments in the Particle Irradiation Data Ensemble (PIDE) dataset (Friedrich et al., 2021). To enrich the physical description beyond conventional macroscopic parameters, we augment the dataset by simulating microdosimetric spectra using the open-source TOPAS toolkit (Perl et al., 2012; Faddegon et al., 2020), and nanodosimetric distributions following the approach described in Grosswendt et al. (2014). The importance of considering multiple spatial scales in radiobiological modeling has also been recently highlighted in Friedrich et al. (2018), where the authors conducted a model-based investigation of how different scales are incorporated into mathematical formulations. Our goal is conceptually similar, that is, understanding the interplay between scales, but we approach it from a fundamentally different perspective. Instead of imposing assumptions through predefined mathematical structures, we develop a DL model that is inherently transparent, allowing interpretation of its decision-making process. This design ensures that no bias is introduced during model construction; rather, the contributions of different scales emerge directly from the data.

We train the model to predict cell survival following irradiation with protons and carbon ions, which is the most widely studied biological endpoint in radiotherapy and particle therapy and is supported by decades of experimental evidence. Cell survival is particularly relevant because it reflects the cumulative effects of processes operating across multiple scales, from DNA damage induction at the nanometer scale to cellular repair dynamics to population-level outcomes. By considering different particle types and clinically relevant energy ranges, our approach enables investigation of how physical and biological scales interact under varying irradiation conditions, providing new insights into the multiscale nature of radiation response.

It is important to emphasize that our primary aim is not to introduce a more accurate predictive model, but rather to investigate interpretability in multiscale radiobiological modeling. Although our DL framework achieves state-of-the-art performance in predicting cell survival, the focus is on exploring how the model reaches its decisions and discerning the relative contributions of different spatial scales of energy deposition. Whereas conventional mathematical models impose scale dependencies through their structure, our transparent DL model allows such relationships to emerge organically from the data, free from structural bias. This enables a clear analysis of nanodosimetric, microdosimetric, and macroscopic descriptors and how they jointly affect cell survival.

In addition to modeling survival curves, we concentrate on three biologically and clinically significant endpoints. First, the dose to 10% survival (*D*_10_), historically the benchmark for calculating relative biological effectiveness (RBE) in particle therapy (Valentin, 2003). RBE is formally defined as


$$\begin{array}{l} \text{RBE}=\frac{D_{\text{ref}}}{D_{\text{test}}}{|}_{\text{same biological effect}} \end{array}$$ (1)

where *D*_ref_ is the dose of reference radiation (e.g., low-LET X-rays), and *D*_test_ is the dose of the test radiation (e.g., protons or carbon ions) required to achieve the same biological effect, typically 10% clonogenic survival. RBE remains a cornerstone in treatment planning since it quantifies how much more (or less) effective a radiation quality is relative to photons, directly guiding dose prescription and clinical outcomes. Radiation quality itself is determined by the fluence spectrum of charged particles at the location of interest, which stochastically specifies the types and energies of particles and thus the spatial pattern of energy deposition (Missiaggia, 2024; Goodhead, 2013). Crucially, the characterization of radiation quality is intimately linked to the size of the target volume over which energy deposition is evaluated: different target sizes, ranging from nanometric DNA segments to micrometric cell nuclei and subnuclear domains, yield different physical descriptions of radiation quality (Villegas et al., 2018; Friedrich et al., 2018; Missiaggia, 2024). Identifying the most biologically relevant target size remains an open question, as no definitive consensus has emerged on the precise shape and dimensions of the critical volume (Missiaggia, 2024). The present work aims to address this issue by systematically investigating the dependence of the predicted biological endpoints on the target size.

Second, we consider the initial slope of the survival curve (the α-region), reflecting intrinsic radiosensitivity and repair mechanisms. It is worth stressing that the term low dose is used here in a sense distinct from its conventional meaning in radioprotection, where doses below 0.1 Gy are typically considered low (Goodhead, 1992). At such dose levels, cell survival experiments are affected by substantial experimental uncertainty, rendering reliable inference difficult. For this reason, we define the low-dose regime as extending up to 1 Gy. Within this range, survival probability and, consequently, attention are evaluated at four discrete dose points (0.25, 0.5, 0.75, and 1 Gy), and the overall low-dose attention is taken as the average across these values. This choice provides a more stable estimate while remaining within the approximately linear region of the survival curve. It is also important to highlight that the low-dose endpoint has long been central to radiobiology, particularly in the assessment of normal tissue response. The definition adopted here is intended to enable a robust and meaningful evaluation of attention for this specific, clinically relevant endpoint.

Finally, we evaluate survival at 2 Gy, which aligns with the conventional fractional dose in radiotherapy, providing a standard benchmark both for modality comparison and for biological modeling.

By combining predictive accuracy, interpretability, and clinically meaningful endpoints, our framework offers a novel lens for understanding how different scales of energy deposition impact biological outcomes. This not only validates model performance but also advances mechanistic insights essential for optimizing particle therapy and personalizing treatment protocols.

## 2 Materials and methods

### 2.1 PIDE dataset

The model was trained and validated using data from the Particle Irradiation Data Ensemble (PIDE, version 3.2) (Friedrich et al., 2021), which includes cell survival experiments for both protons and carbon ions. The PIDE provides detailed specifications of the radiation fields used in these experiments. Leveraging this information, we reproduced the experimental conditions through Monte Carlo (MC) simulations to extract additional physical descriptors, including nanodosimetric and microdosimetric quantities.

Cell survival following irradiation was modeled using the linear-quadratic (LQ) formalism, which expresses survival as


$$\begin{array}{l} S\left(D\right)=\exp\left(-\alpha D-\beta D^{2}\right), \end{array}$$

where *D* is the absorbed dose, and α and β are two parameters that depend on both the biological system and the radiation field. In the following, we denote by α_*X*_ and β_*X*_ the parameters corresponding to the reference radiation. The ratio α/β denotes the dose at which these two components are equal and is widely used to characterize tissue radiosensitivity. This parameter is fundamental in radiotherapy for guiding fractionation schemes and comparing biological effects across radiation qualities. Following previous works (Cordoni et al., 2023; Pfuhl et al., 2022), the DL model was trained on parameters derived from an exponential LQ fit of the survival curves rather than raw survival fractions. This choice addresses the substantial variability observed in reported survival data across experiments, which can introduce noise and bias. To ensure robust curve fitting, experiments reporting fewer than three measurement points were excluded, as at least three data points are required to estimate LQ parameters meaningfully.

The dataset, comprising 358 experiments, was split into three folds at the experiment level (119, 119, and 120 experiments, respectively), ensuring that all data from a single experiment were assigned to the same fold and that experiments in the validation split had never been seen during training. This strategy ensures a more robust evaluation of the model's generalization capability. This strategy prevents data leakage across folds and allows for a more robust evaluation of generalization. The model was trained iteratively on two folds and validated on the remaining fold, cycling through all three combinations. This approach enables out-of-sample validation across the entire dataset, which is particularly important given the limited data available. A standard train–test split would not provide sufficient robustness for the test set under these constraints. All results reported in this work refer to performance on the three validation sets.

A logarithmic transformation of the surviving fraction data is applied to smooth the training. Further, the Box-Cox transformation of LET and microdosimetric values is applied to stabilize variance and improve normality.

For this study, 320 experiments are selected, covering the full available PIDE cohort of proton and carbon-ion irradiations. This choice allows us to consider protons from energy 0.67 MeV and LET 33 keV/μm to energy 76 MeV and LET 0.9 keV/μm; while experiments with carbon ions go from energy 1.69 MeV/u and LET 576 keV/μm to energy 440 MeV/u and LET 10.5 keV/μm.

In the following, we will compare the developed DL model with two standard models used in the clinics, the Microdosimetric Kinetic Model (MKM) (Inaniwa and Kanematsu, 2018) and the Local Effect Model (LEM) version III (Elsässer et al., 2008). As done in previous work (Cordoni et al., 2023), the model's implementation and parameters are reported in (Manganaro et al., 2018). The comparison is performed using the HSG and V79 cell lines, which are the ones for which the LEM and MKM have been extensively calibrated and validated.

### 2.2 Nanodosimetry

Nanodosimetry seeks to characterize the stochastic nature of energy deposition at nanometer scales by analyzing ionization cluster-size distributions (ICSD) within target volumes comparable to segments of cellular DNA (Rabus, 2011; Grosswendt, 2007). This approach reveals how charged particles produce localized clusters of ionizations that are critical in causing complex DNA damage–lesions that are often irreparable and can lead to cell death or mutation. In our work, we quantify this effect using the complementary cumulative distribution function


$$\begin{array}{l} \bar{F}\left(n\right)=\underset{\nu >n}{\sum }p\left(\nu \right), \end{array}$$

where *p*(ν) is the probability of observing ν ionizations in the nanometer target volume. $\bar{F}\left(n\right)$ captures the probability of observing ionization clusters larger than size *n*.

By focusing on $\bar{F}\left(n\right)$, we provide a sensitive descriptor of ionization events likely to induce complex DNA damage. Larger cluster sizes correlate with increased biological effect, such as double–strand breaks, resulting in a decrease in cell survival probability, which cannot be adequately captured by average descriptors like linear energy transfer (LET). This makes the approach especially relevant across particle types and energies, as large clusters are more frequent in high–LET radiation such as carbon ions. Overall, nanodosimetry provides a window into the microscopic structure of radiation tracks and their biological impact. Quantifying the frequency of ionization clusters of a given size yields mechanistic insight into radiation-induced DNA lesions, thereby enhancing the interpretability and predictive power of radiobiological models for cell survival. It has been further recently shown that $\bar{F}\left(n\right)$, for *n* = 1, 2, 3, correlates with survival probability (Conte et al., 2024). Furthermore, experimental studies have demonstrated that the cumulative probability of observing at least two or three ionizations in nanometer-sized volumes is proportional to the inactivation cross-section at defined survival levels across different cell lines and radiation qualities (Selva et al., 2019). Advanced Monte Carlo track-structure simulations have also validated these distributions and their connection to biological endpoints, supporting the use of cluster–dose metrics in treatment planning (Faddegon et al., 2023; Ortiz et al., 2025; Hilgers et al., 2024).

In this study, ionization cluster size distributions were simulated using the Monte Carlo code MC-Startrack (De Nardo et al., 2002; Grosswendt et al., 2014). Different particles at different energies were directed toward a spherical propane target volume (0.15 mm diameter, 300 Pa). Following the scaling approach described in Grosswendt (2004), this configuration corresponds to a water-equivalent target with an effective diameter of approximately 1 nm in terms of ionization cluster size distributions.

### 2.3 Microdosimetry

Microdosimetry aims to describe the stochastic nature of energy deposition at the micrometer scale, which is relevant to cellular and subcellular structures such as nuclei and chromatin domains. Unlike nanodosimetry, which focuses on ionization clusters within DNA-sized volumes, microdosimetry characterizes the distribution of energy imparted to volumes comparable to whole cells or organelles, providing insight into the probability of inducing clustered DNA damage and its repair dynamics. This approach is essential for understanding biological endpoints such as clonogenic survival, as it bridges the gap between track-structure physics and radiobiological response at the cellular level [International Commission on Radiation Units and Measurements (ICRU), 2016; Rossi and Zaider, 1996]. In this study, microdosimetric quantities were simulated using the TOPAS Monte Carlo toolkit (version 3.9) (TOPAS MC Inc. SLAC National Accelerator Laboratory - Menlo Park, California, USA) (Perl et al., 2012), which has been validated against experimental measurements for a variety of radiation qualities (Zhu et al., 2019; Missiaggia et al., 2023; Burigo et al., 2014). Radiation fields were generated to replicate the experimental conditions reported in PIDE, with particles directed from an environmental source toward the center of the simulated geometry. Energy deposition was scored in a water sphere of 1μm diameter, representing a typical cell nucleus. The primary descriptors considered are the frequency-mean lineal energy ȳ_*F*_ and dose-mean lineal energy ȳ_*D*_, defined as:


$$\begin{array}{l} {ȳ}_{F}=\int_{0}^{\infty }yf\left(y\right)dy,\;{ȳ}_{D}=\frac{1}{{ȳ}_{F}}\int_{0}^{\infty }y^{2}f\left(y\right)dy, \end{array}$$

where *f*(*y*) is the probability density function of lineal energy *y*, expressed in keV/μm. The quantity ȳ_*F*_ represents the average energy deposition per unit length for individual events, while ȳ_*D*_ weights higher-energy events more strongly, making it particularly relevant for assessing biological effectiveness. These metrics have been historically linked to RBE and cell survival, as they capture the heterogeneity of energy deposition at the micrometer scale, which influences the induction of complex DNA damage and subsequent repair processes [International Commission on Radiation Units and Measurements (ICRU), 2016; Rossi and Zaider, 1996].

By incorporating microdosimetric descriptors alongside nanodosimetric quantities, we enable a multiscale characterization of radiation quality, providing a more comprehensive basis for predicting biological outcomes such as cell survival. This integration is crucial because different spatial scales, nanometers for DNA damage induction and micrometers for cellular energy deposition, jointly determine the probability of cell inactivation. Microdosimetry is currently implemented in clinical carbon ion therapy, particularly in Japan, where it serves as the basis for the MKM used to calculate relative biological effectiveness (RBE) for treatment planning (Hawkins, 1994; Inaniwa et al., 2010; Bellinzona et al., 2021).

### 2.4 Deep Learning with attention mechanism

The developed model is based on *TabNet* (Arik and Pfister, 2021), a DL architecture specifically designed for tabular data. It employs a sequential attention mechanism that enables adaptive feature selection at each decision step, achieving high predictive performance while providing intrinsic interpretability.

The model operates through a sequence of *T* decision steps. At each step, an attentive transformer produces a sparse feature selection mask that determines which input features are used. The masked features are then processed by a feature transformer, composed of fully connected layers with Gated Linear Units (GLUs), to generate higher-level representations. The feature transformer is composed of shared and decision-step-specific blocks, enabling parameter sharing across steps while allowing step-wise specialization. At each decision step, an attentive transformer computes a sparse feature mask using Sparsemax, conditioned on both the transformed features from the previous step and a feature prior that discourages repeated feature usage across steps. This prior is updated iteratively, promoting diverse feature selection and enhancing interpretability. The masked input is then processed by the feature transformer, producing both decision outputs and representations for subsequent steps. A sparsity regularization term based on the entropy of the attention masks is added to the training objective.

The architecture consists of four main components: (i) Feature Transformer, (ii) Attentive Transformer, (iii) Feature Prior and Decision Steps, and (iv) Sparse Feature Selection and Regularization.

#### 2.4.1 Feature transformer

Let


$$\begin{array}{l} \text{X}\in {\mathbb{R}}^{n\times d} \end{array}$$

denote the input matrix, where *n* is the number of samples and *d* is the number of features. The feature transformer maps masked input features into higher-level representations using a stack of fully connected layers followed by GLU. It consists of a set of shared layers, common to all decision steps, and step-specific layers that allow specialization at each step.

A generic transformation can be written as


$$\begin{array}{l} \text{H}=\text{GLU}\left(\text{X}{\text{W}}_{1}+{\text{b}}_{1}\right),\;{\text{H}}^{\prime }=\text{GLU}\left(\text{H}{\text{W}}_{2}+{\text{b}}_{2}\right), \end{array}$$

where **W**_1_, **W**_2_ are learnable weight matrices and **b**_1_, **b**_2_ are bias vectors.

The Gated Linear Unit is defined as


$$\begin{array}{l} \text{GLU}\left(\text{Z}\right)={\text{Z}}_{a}⊙\sigma \left({\text{Z}}_{b}\right), \end{array}$$

where **Z** ∈ ℝ^*n*×2*h*^ is split into two equal parts ${\text{Z}}_{a},{\text{Z}}_{b}\in {\mathbb{R}}^{n\times h}$, ⊙ denotes element-wise multiplication, and σ(·) is the sigmoid activation function. This gating mechanism enables adaptive control of information flow and improves model stability.

#### 2.4.2 Attentive transformer and feature prior

At each decision step *t*, the attentive transformer computes a sparse feature selection mask **M**^(*t*)^∈ℝ^*n*×*d*^ using the Sparsemax activation function. The mask is conditioned on the transformed features from the previous step and a feature prior **P**^(*t*)^, which controls feature reuse across decision steps:


$$\begin{array}{l} {\text{M}}^{\left(t\right)}=\text{Sparsemax}\left({\text{A}}^{\left(t\right)}⊙{\text{P}}^{\left(t\right)}\right), \end{array}$$

where **A**^(*t*)^ denotes the output of the attentive transformer and ⊙ represents element-wise multiplication.

The feature prior is initialized as **P**^(1)^ = **1** and updated after each decision step according to


$$\begin{array}{l} {\text{P}}^{\left(t+1\right)}=\gamma \left({\text{P}}^{\left(t\right)}-{\text{M}}^{\left(t\right)}\right),\;\gamma >1, \end{array}$$

thereby discouraging repeated selection of the same features and promoting diversity across decision steps.

The Sparsemax function projects its input onto the probability simplex:


$$\begin{array}{l} \text{Sparsemax}\left(\text{z}\right)=\arg\underset{\text{p}\in {\text{Δ}}^{d}}{\min}||\text{p}-\text{z}{||}_{2}^{2}, \end{array}$$

where


$$\begin{array}{l} \Delta^{d}=\left\{\text{p}=\left(p_{1},\ldots ,p_{d}\right)\in {\mathbb{R}}^{d}|\text{p}\geq 0,\;\overset{d}{\underset{j=1}{\sum }}p_{j}=1\right\}. \end{array}$$

Unlike Softmax, Sparsemax produces sparse probability distributions, enabling explicit feature selection.

#### 2.4.3 Decision steps and aggregation

At decision step *t*, the input features are masked as


$$\begin{array}{l} {\text{X}}^{\left(t\right)}={\text{M}}^{\left(t\right)}⊙\text{X}, \end{array}$$

and passed through the feature transformer to produce a latent representation


$$\begin{array}{l} {\text{H}}^{\left(t\right)}=\text{FeatureTransformer}\left({\text{X}}^{\left(t\right)}\right). \end{array}$$

Each step yields a decision output *P*^(*t*)^, and the final model prediction is obtained by aggregating all decision step outputs:


$$\begin{array}{l} P=\overset{T}{\underset{t=1}{\sum }}P^{\left(t\right)}. \end{array}$$

#### 2.4.4 Sparse regularization

To further encourage sparse and interpretable feature selection, a regularization term based on the entropy of the attention masks is included


$$\begin{array}{l} L_{\text{reg}}=\frac{\lambda }{T}\overset{T}{\underset{t=1}{\sum }}\overset{d}{\underset{j=1}{\sum }}M_{j}^{\left(t\right)}\log\left(M_{j}^{\left(t\right)}+\epsilon \right), \end{array}$$

where λ controls the regularization strength and ϵ is a small constant for numerical stability. This term penalizes dense feature usage, enhancing interpretability by encouraging the model to focus on a limited subset of features at each decision step.

**Figure 1:**
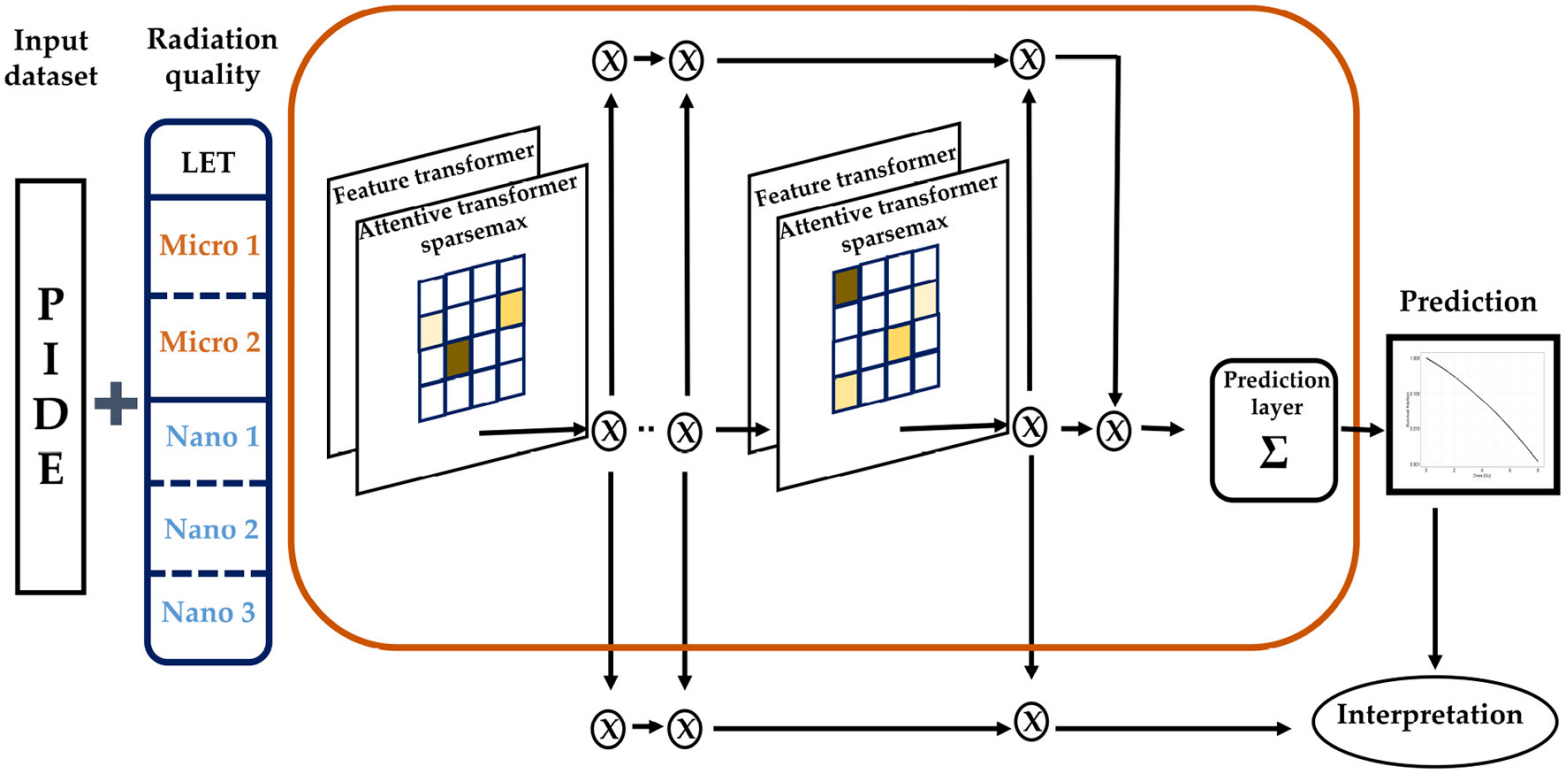
DL model workflow.

Figure 1 illustrates the overall architecture of the model, from dataset construction using PIDE to the sequential attention-based decision steps leading to the final interpretable predictions.

### 2.5 Error analysis and biological endpoints considered

All the selected data from PIDE, where only the LET is reported, are augmented with the nano- and microdosimetric quantities. This dataset also contains the biological parameters of each experiment in terms of the linear-quadratic parameters for photons α_*X*_ and β_*X*_. Performance is assessed using Mean Absolute Percentage Error (MAPE) and Root Mean Square Error (RMSE). The latter is defined for each SF experiment as


$$\begin{array}{l} RMSE_{i}:=\sqrt{\frac{1}{N_{D}}\underset{D}{\sum }{\left(\log\left({Ŝ}_{i}\left(D\right)\right)-\log\left(S_{i}\left(D\right)\right)\right)}^{2}}, \end{array}$$

where *N*_*D*_ is the number of doses measured in the *i*-th experiment, while Ŝ_*i*_ and *S*_*i*_ are the cell survivals predicted and measured, respectively. On the other hand, given N observations, true values *y*_*i*_, and predictions ŷ_*i*_, MAPE can be defined as


$$\begin{array}{l} \text{MAPE}:=\frac{1}{N}\overset{N}{\underset{i=0}{\sum }}|\frac{\text{RB}{\text{E}}_{i}-\text{R}{\hat{\text{B}}\text{E}}_{i}}{\text{RB}{\text{E}}_{i}}|\times 100. \end{array}$$

This value is computed from the predictions of RBE_10_, defined in Equation 1, where the biological endpoint corresponds to a survival probability of 10%.

Three different biological endpoints are considered throughout the paper to study the attention masks: (i) the dose corresponding to 10% survival probability, (ii) dose 2 Gy, and (iii) the low dose limits. Regarding this last endpoint, we consider the average of the attention at doses 0.25, 0.5, 0.75, and 1 Gy. All importance values reported throughout the manuscript are normalized by the number of experiments in a given radiation field condition, ensuring that conditions with more replicates do not disproportionately influence the final importance scores.

Importance curves against the energy of the beam for protons and carbon ions are generated using Locally Estimated Scatterplot Smoothing (LOESS) (Cleveland, 1979), a non-parametric method for smoothing a series of data, and also constructing confidence intervals around the curve.

Analyses were conducted with Rversion4.4.2 with torch (0.16.0), tidyverse (2.0.0), rUM (2.2.0), and gtsummary (2.4.0) packages (R Core Team, 2024; Collobert et al., 2002; Wickham, 2023; Wickham et al., 2019; Wickham, 2016; Balise et al., 2025).

## 3 Results

**Figure 2:**
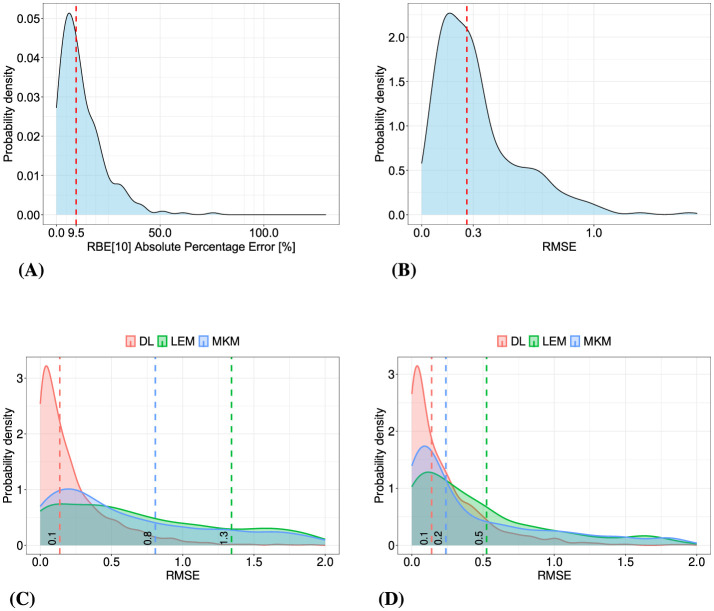
Model performance. **(A)** Absolute percentage error. **(B)** RMSE. **(C)** RMSE comparison for the V79 cells. **(D)** RMSE comparison for the HSG cells. **(A)** Absolute percentage error across the whole PIDE dataset. The red dotted line indicates the average value. **(B)** RMSE distribution across the whole PIDE dataset. The red dotted line indicates the average value. **(C)** RMSE for the V79 cell line comparing our model, LEM, and MKM. The colored dotted lines indicate the average values. **(D)** RMSE for the HSG cell line comparing our model, LEM, and MKM. The colored dotted lines indicate the average values.

The accuracy of the RBE_10_ model predictions can be evaluated using the absolute percentage error distribution (Figure 2A, with a MAPE of 9.5%. Figure 2B also shows the evaluation in terms of RMSE distribution, with a mean of 0.3. In Figures 2C, D, we compare the results of our model with the MKM (Kase et al., 2007) and LEM (Russo et al., 2011) on survival data coming from V79 and HSG cell lines, which are the most represented in the whole dataframe. The multiscale DL model shows in both cases the lowest RSME around 0.1. The performance of MKM is assessed at 0.8 and 0.2 for V79 and HSG, respectively, while the corresponding values for LEM are 1.3 and 0.5.

**Figure 3:**
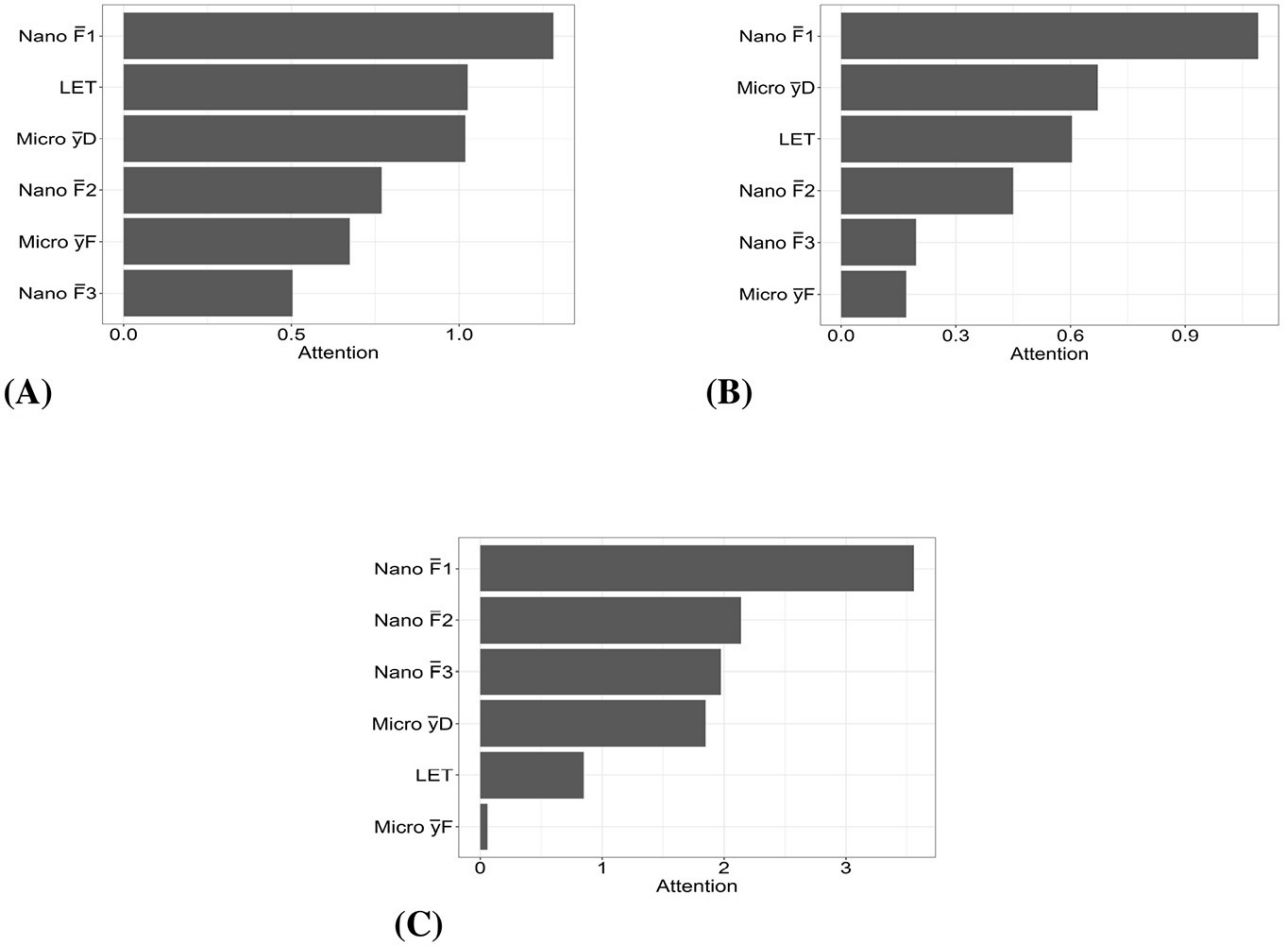
Global attention for the three endpoints considered. **(A)** Dose corresponding to 10% survival. **(B)** 2 Gy. **(C)** Low-dose limit. **(A)** Global attention evaluated at the dose corresponding to 10% survival, extracted from all attention steps across the entire dataset. **(B)** Global attention evaluated at a dose of 2 Gy, extracted from all attention steps across the entire dataset. **(C)** Global attention evaluated at the low-dose limit, extracted from all attention steps across the entire dataset.

We assessed the contribution of each feature using the built-in interpretability of the attention mechanism at each prediction step. Global feature importance is summarized in Figure 3, with detailed results for *D*_10_ in Figure 3A, for survival at 2 Gy in Figure 3B, and for the low-dose limit in Figure 3D. Across all endpoints, the nanodosimetric descriptor $\bar{F}\left(1\right)$ consistently exhibits the highest attention, indicating its dominant role in survival prediction. For *D*_10_ and 2 Gy, attention is more evenly distributed, with ȳ_*D*_ and LET emerging as the most influential features after $\bar{F}\left(1\right)$. In contrast, the low-dose limit shows a clear predominance of nanodosimetric metrics, with only ȳ_*D*_ approaching the same level of importance. These results highlight the varying relevance of physical descriptors across biological endpoints, with nanodosimetry playing a particularly critical role in the low-dose region.

**Figure 4:**
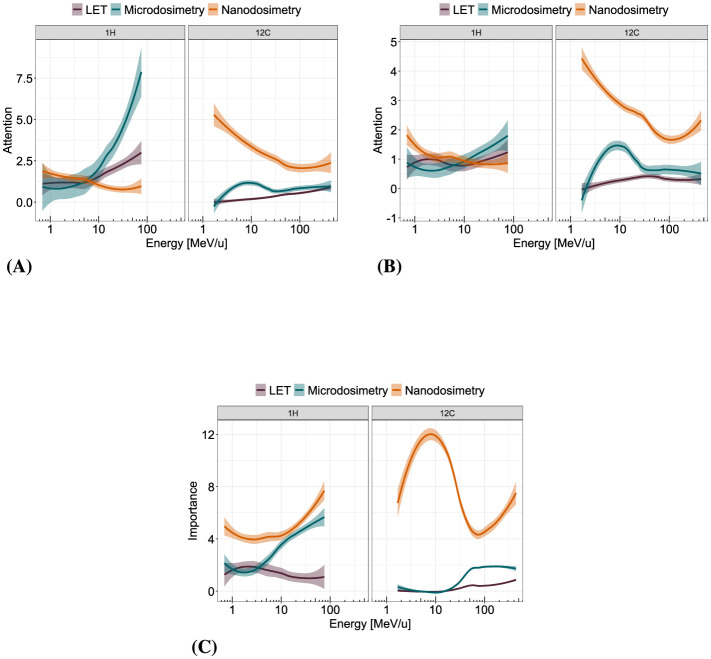
Attention as a function of beam energy for protons and carbon ions for the three endpoints considered. **(A)** Dose corresponding to 10% survival. **(B)** 2 Gy. **(C)** Low-dose limit. **(A)** Attention to LET, microdosimetric, and nanodosimetric features evaluated at the dose corresponding to 10% survival as a function of beam energy for protons and carbon ions. Shaded areas indicate 95% confidence intervals obtained with the LOESS method. **(B)** Attention to LET, microdosimetric, and nanodosimetric features evaluated at 2 Gy as a function of beam energy for protons and carbon ions. Shaded areas indicate 95% confidence intervals obtained with the LOESS method. **(C)** Attention to LET, microdosimetric, and nanodosimetric features evaluated at the low-dose limit as a function of beam energy for protons and carbon ions. Shaded areas indicate 95% confidence intervals obtained with the LOESS method.

While global importance offers a broad overview, our goal was to achieve a deeper understanding by analyzing local importance, which captures condition-specific contributions to model predictions. Figure 4 illustrates attention as a function of beam energy, separated for protons and carbon ions, with detailed results for *D*_10_ in Figure 4A, survival at 2 Gy in Figure 4B, and the low-dose limit in Figure 4C. Different spatial scales are represented by distinct colors. For protons, in the case of *D*_10_ and survival at 2 Gy, microdosimetry and LET exhibit similar trends, generally increasing with energy, whereas nanodosimetry shows the opposite behavior. Microdosimetry becomes significantly more important at higher proton energies for *D*_10_, while attention is more uniformly distributed for survival at 2 Gy. In contrast, LET has minimal importance for carbon ion irradiation. However, its trend remains broadly similar to microdosimetry, except for a small peak around 10 MeV/u in the attention to microdosimetric features. Nanodosimetry shows a global decrease with increasing beam energy for carbon ions, but its relative importance compared to higher-level scales is striking–approximately three times that for protons. For the low-dose limit, nanodosimetry emerges as the dominant contributor for both protons and carbon ions, with its attention in carbon ions surpassing all other scales. LET maintains a nearly constant importance for both particle types, whereas microdosimetry shows an increasing trend with energy. Nanodosimetry, on the other hand, reveals an upward trend at higher proton energies and a complex pattern for carbon ions, characterized by a pronounced peak near 10 MeV/u. The shaded regions around the smoothed importance curves represent 95% confidence intervals, reflecting uncertainty in the mean predicted importance across different radiosensitivities of the cell lines included in the dataset. The small negative attention values observed in the very low energy region should be interpreted as numerical artifacts of the LOESS smoothing procedure, which cannot fully accommodate the sharp local variations of the data, and are therefore physically consistent with zero.

**Figure 5:**
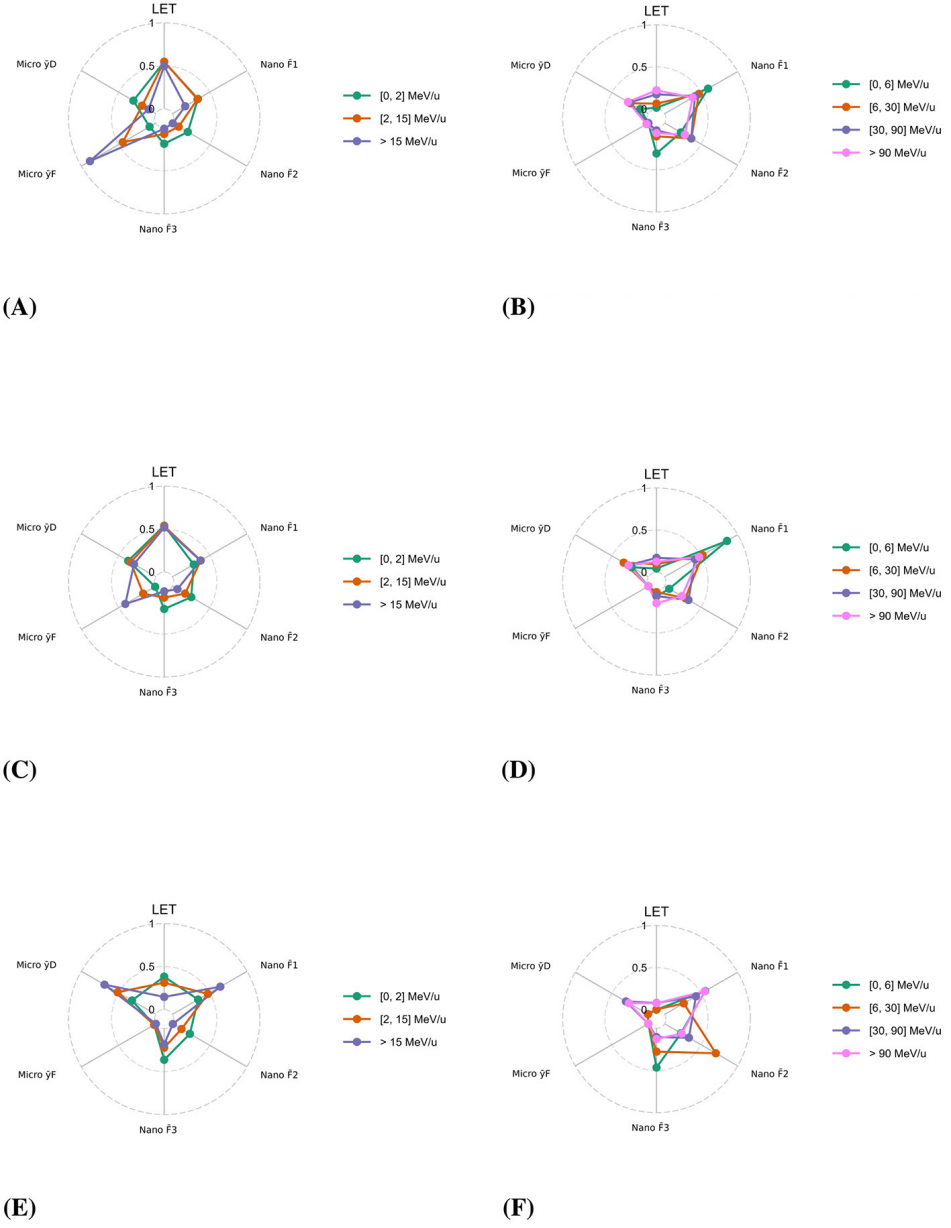
Spider plots showing model attention for protons and carbon ions across the three endpoints considered. **(A, B)** Dose corresponding to 10% survival. **(C, D)** 2 Gy. **(E, F)** Low-dose limit. **(A)** Spider plot showing attention for protons at the dose corresponding to 10% survival. Different colors represent different energy bins. **(B)** Spider plot showing attention for carbon ions at the dose corresponding to 10% survival. Different colors represent different energy bins. **(C)** Spider plot showing attention for protons at a dose of 2 Gy. Different colors represent different energy bins. **(D)** Spider plot showing attention for carbon ions at a dose of 2 Gy. Different colors represent different energy bins. **(E)** Spider plot showing attention for protons at the low-dose limit. Different colors represent different energy bins. **(F)** Spider plot showing attention for carbon ions at the low-dose limit. Different colors represent different energy bins.

We further analyzed feature attention using spider plots, grouping experiments by particle energy as indicated in the color legend. Figures 5A–E show results for protons at *D*_10_, 2 Gy, and the low-dose limit, with attention weights normalized to the total for each endpoint. Different colors represent energy bins, illustrating how feature importance varies with beam energy. For protons, microdosimetric contributions increase with energy, driven primarily by ȳ_*F*_, while ȳ_*D*_ remains comparable at lower energies for *D*_10_ and 2 Gy. In the low-dose limit, nanodosimetry, particularly $\bar{F}\left(1\right)$, dominates across all energies, with only ȳ_*D*_ approaching similar importance.

The same analysis for carbon ions (Figures 5B–F) confirms the overall dominance of nanodosimetric descriptors over other scales. At higher energies, microdosimetry and LET gain relative importance, whereas at lower energies $\bar{F}\left(1\right)$ and $\bar{F}\left(3\right)$ remain the most influential for *D*_10_ and 2 Gy. In the low-dose region, nanodosimetry again prevails, with attention shifting toward $\bar{F}\left(1\right)$ as energy increases and decreasing for $\bar{F}\left(3\right)$; $\bar{F}\left(2\right)$ shows a peak at intermediate energies.

**Figure 6:**
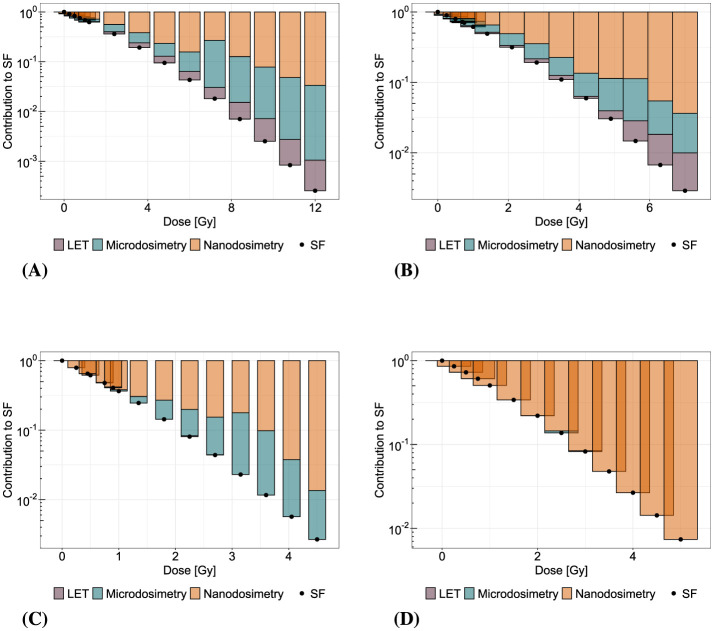
Contribution of the different spatial scales (color-coded) to survival fraction calculations for carbon-ion irradiation of the V79 cell line at four LET values. **(A)** 22 keV/μm, **(B)** 41 keV/μm, **(C)** 142 keV/μm, **(D)** 360 keV/μm. **(A)** Contribution of the different spatial scales (color-coded) to survival fraction calculations for carbon-ion irradiation of the V79 cell line at LET 22 keV/μm. **(B)** Contribution of the different spatial scales to survival fraction calculations for carbon-ion irradiation of the V79 cell line at LET 41 keV/μm. **(C)** Contribution of the different spatial scales to survival fraction calculations for carbon-ion irradiation of the V79 cell line at LET 142 keV/μm. **(D)** Contribution of the different spatial scales to survival fraction calculations for carbon-ion irradiation of the V79 cell line at LET 360 keV/μm.

**Figure 7:**
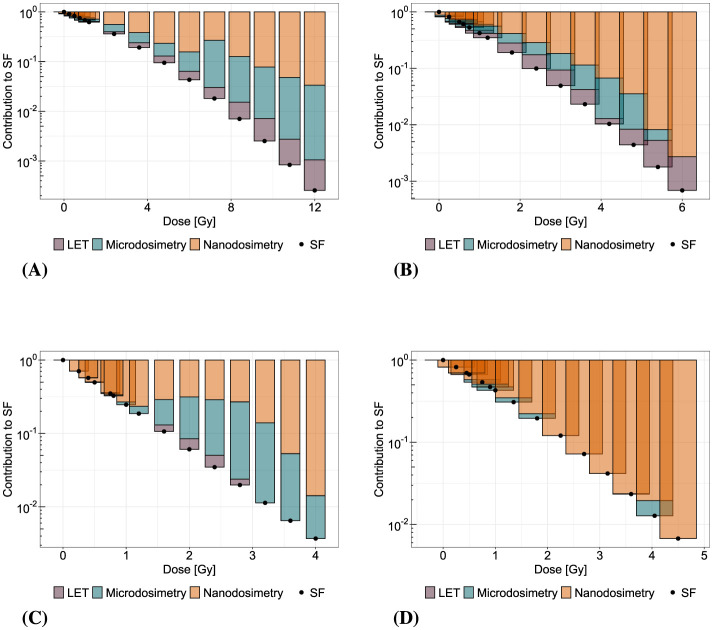
Contribution of the different spatial scales (color-coded) to survival fraction calculations for carbon-ion irradiation of the HSG cell line at four LET values. **(A)** 23 keV/μm, **(B)** 40 keV/μm, **(C)** 144 keV/μm, **(D)** 467 keV/μm. **(A)** Contribution of the different spatial scales (color-coded) to survival fraction calculations for carbon-ion irradiation of the HSG cell line at LET 23 keV/μm. **(B)** Contribution of the different spatial scales to survival fraction calculations for carbon-ion irradiation of the HSG cell line at LET 40 keV/μm. **(C)** Contribution of the different spatial scales to survival fraction calculations for carbon-ion irradiation of the HSG cell line at LET 144 keV/μm. **(D)** Contribution of the different spatial scales to survival fraction calculations for carbon-ion irradiation of the HSG cell line at LET 467 keV/μm.

Figure 6 shows the contribution of different spatial scales to the prediction of the surviving fraction values for the V79 cell line. All experiments are taken from Furusawa et al. (2000). We consider data from one single experiment with carbon ions irradiation corresponding to four different LET values: Figure 6A 22 keV/μm, Figure 6B 41 keV/μm, Figure 6C 142 keV/μm, and Figure 6D 360 keV/μm. The predicted SF points are represented by black dots, while colored bars show the relative contributions of different scales to overall survival. We show the same results in Figure 7 for the HSG cell line. In this case the LET values are Figure 7A 23 keV/μm, Figure 7B 40 keV/μm, Figure 7C 144 keV/μm and Figure 7D 467 keV/μm.

**Figure 8:**
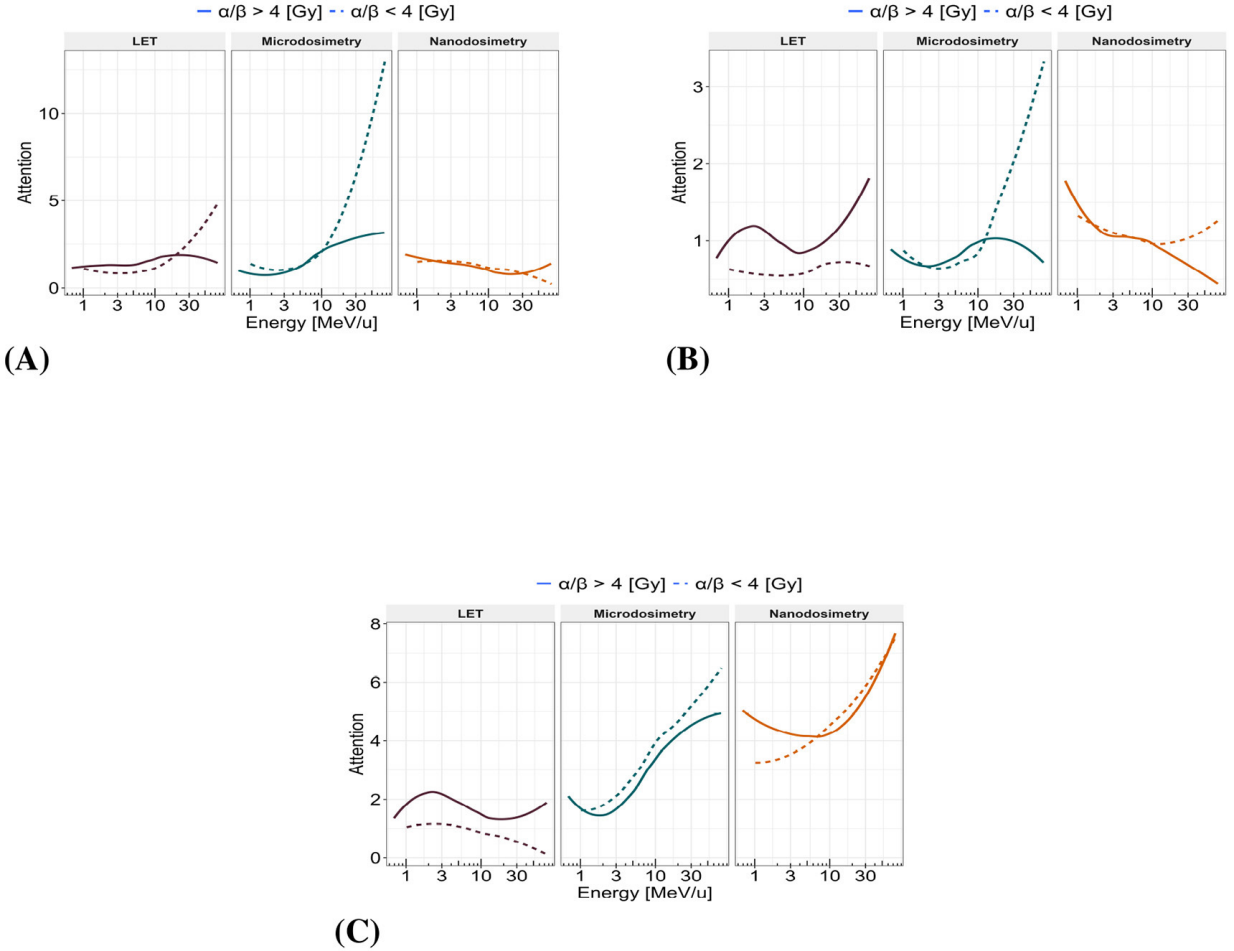
Attention as a function of beam energy for protons, grouped by cell-line radiosensitivity expressed in terms of the α_*X*_/β_*X*_ ratio. **(A)** Dose corresponding to 10% survival. **(B)** 2 Gy. **(C)** Low-dose limit. **(A)** Attention to LET, microdosimetric, and nanodosimetric features evaluated at the dose corresponding to 10% survival as a function of beam energy for protons. Linetype indicates different cell-line radiosensitivities expressed by the α_*X*_/β_*X*_ ratio. **(B)** Attention to LET, microdosimetric, and nanodosimetric features evaluated at 2 Gy as a function of beam energy for protons. Linetype indicates different cell-line radiosensitivities expressed by the α_*X*_/β_*X*_ ratio. **(C)** Attention to LET, microdosimetric, and nanodosimetric features evaluated at the low-dose limit as a function of beam energy for protons. Linetype indicates different cell-line radiosensitivities expressed by the α_*X*_/β_*X*_ ratio.

Figure 8 illustrates attention as a function of particle energy, separated into panels for LET, microdosimetry, and nanodosimetry for protons at *D*_10_ (Figure 8A), 2 Gy (Figure 8B), and the low-dose limit (Figure 8C). Line styles distinguish cell lines by radiosensitivity, following the convention in particle therapy: solid lines correspond to α_*X*_/β_*X*_>4 Gy, while dashed lines represent α_*X*_/β_*X*_ < 4 Gy.

For *D*_10_ and 2 Gy, LET and microdosimetry show relatively stable attention for highly radiosensitive cell lines (α_*X*_/β_*X*_>4 Gy), but increase with energy for less sensitive lines (α_*X*_/β_*X*_ < 4 Gy). Nanodosimetry exhibits minimal differences between cell types, with only a slight decrease in attention at high energies for α_*X*_/β_*X*_ < 4 Gy at 2 Gy.

In the low-dose limit, attention generally rises across all scales, with nanodosimetry remaining dominant. LET shows an almost constant trend, consistently higher for α_*X*_/β_*X*_>4 Gy. Microdosimetry follows a similar increasing pattern, while nanodosimetry demonstrates a strong upward trend with energy, with α_*X*_/β_*X*_>4 Gy receiving slightly more attention at low energies.

## 4 Discussion

Radiation response in particle therapy spans multiple biological scales, from clustered DNA damage at the nanometer level, through disruption of chromosomal and cellular structures at the micrometer scale, to normal–tissue and tumor responses at the macroscopic level. Each scale has inspired distinct modeling approaches that are widely used, yet typically applied predominantly using a single scale.

Nanodosimetry characterizes the stochastic nature of energy deposition at nanometer scales by analyzing ionization cluster-size distributions (ICSD) within DNA-sized volumes (Rabus, 2011; Grosswendt, 2007). Large clusters are strongly associated with complex, often irreparable DNA lesions, making nanodosimetric descriptors such as cumulative probabilities $\bar{F}\left(n\right)$ valuable for linking track structure to biological severity. Recent computational and experimental studies have demonstrated how these metrics can inform treatment planning, for example, in spread-out Bragg peak (SOBP) fields for protons and carbon ions (Ramos-Méndez et al., 2021; Selva et al., 2019; Faddegon et al., 2023).

Microdosimetry addresses energy deposition at the micrometer scale, historically providing the bridge between physical dose and biological effect through probabilistic quantities such as lineal energy (*y*) [Rossi and Zaider, 1996; International Commission on Radiation Units and Measurements (ICRU), 2016]. These concepts underpin the MKM, which remains the clinical standard for calculating RBE in carbon ion therapy, particularly in Japan (Kanai et al., 1999). Microdosimetric descriptors like ȳ_*F*_ and ȳ_*D*_ are routinely used to characterize radiation quality and optimize treatment plans (Missiaggia et al., 2023; Inaniwa et al., 2010; Bellinzona et al., 2021).

At the macroscopic level, LET has long served as a practical descriptor of radiation quality. High-LET radiation, such as carbon ions, produces dense ionization tracks and greater biological impact than low-LET radiation, such as protons or photons. LET optimization strategies exploit this property by modulating beam energies and fluence to increase LET in tumor regions while sparing normal tissue, and recent work has proposed incorporating LET-based objectives into inverse planning (Grupp and et al., 2023; Giantsoudi and et al., 2016).

Together, nanodosimetry, microdosimetry, and LET provide complementary perspectives on radiation quality and biological effect. However, these approaches are almost always applied separately, leaving open the challenge of integrating multiscale information into unified predictive frameworks. Addressing this gap is essential for advancing biologically guided particle therapy.

As a general rule, lower-energy particles tend to be more biologically effective than higher-energy particles due to their higher LET. However, for carbon ions, when LET exceeds approximately 150 keV/μm (corresponding to energies below about 10 MeV/u), the trend reverses: biological effectiveness decreases despite increasing LET. This phenomenon, known as the *overkill effect*, occurs because excessive ionization within a small volume leads to redundant damage that does not further reduce survival (Bellinzona et al., 2021).

The results demonstrate that, to achieve accurate predictions of radiation-induced biological effects, the model integrates information across multiple spatial scales, rather than relying on a single physical descriptor of the radiation field. The combined contributions from these quantities are essential for capturing the full complexity of radiation–matter interactions and subsequent biological responses. This study supports ongoing efforts to incorporate further physical descriptions of radiation quality into treatment-plan optimization. Historically, biological dose optimization in proton therapy has typically assumed a constant RBE of 1.1, whereas for carbon ions, models such as MKM or LEM have been used to guide planning. However, recent work has shifted toward optimizing not just dose but also physical descriptors such as LET, especially in proton therapy, where LET-guided optimization has already been implemented in clinical settings, and in carbon-ion therapy, where LET painting strategies are being explored to enhance biological effectiveness (Deng et al., 2021; Reiners et al., 2025). Beyond LET, there is growing interest in integrating nanodosimetric and microdosimetric quantities into optimization frameworks. For example, cluster-dose optimization for protons and helium has been proposed based on nanodosimetric metrics derived from Monte Carlo track-structure simulations (Facchiano et al., 2025; Faddegon et al., 2023), while carbon-ion plans have been optimized using nanodosimetric quantity-weighted dose approaches (Yang et al., 2024). Similarly, microdosimetric parameters have been shown to provide alternative and effective descriptors of radiation quality in clinical planning, rather than relying solely on LET (Inaniwa et al., 2010; Magini et al., 2025), enabling novel microdosimetry-guided treatment planning strategies. These efforts underscore a movement toward multi-scale, physics-informed optimization that extends beyond conventional biological-dose models. Moreover, it highlights the value of considering multiple spatial scales rather than relying on a single one. More importantly, it emerges a complex pattern that relates different scales, particle type, energy, and cell-line radiosensitivity, corroborating the idea that several factors contribute to the final radiobiological endpoint.

Regarding the accuracy of the proposed multiscale DL model, it shows improved predictive performance compared to the clinical reference models, MKM and LEM, and other approaches. Performance, evaluated using RMSE in Figure 2B, consistently yields lower values for our multiscale model across the data from the V79 and HSG cell lines. These results suggest that the architecture better captures the data's underlying multiscale patterns, leading to more accurate estimates than the reference models.

The comparison between protons and carbon ions highlights both similarities and fundamental differences in their radiobiological behavior. In general, we could say that *D*10 and dose 2 Gy show similar attentions, whereas a different pattern emerges in the low dose limit. Although both protons and carbon ions exhibit comparable qualitative trends in the importance of predictors from all spatial scales against beam energy, the relative importance of nanodosimetric quantities is much higher in the case of carbon ions, as shown in Figure 4. This effect arises from the characteristic energy deposition along tracks of carbon ions, with higher charge and LET compared to protons. The importance of nanodosimetry reflects the much denser ionization patterns and more complex DNA damage produced by heavier ions. The varying importance across energies suggests that different biological mechanisms can contribute differently to cell inactivation for different particles. Further, shaded areas around the LOESS smoothing reflect biological variability. In this study, all this information is condensed in the α_*X*_ and β_*X*_ parameters for cell survival curves from X-ray irradiation. It is interesting to note that LET in carbon ions shows little to no shaded area, indicating that the DL model treated LET as a biological invariant, offsetting other physical descriptions.

A similar observation applies to the low-dose limit (below 1 Gy), where nanodosimetry clearly dominates. This trend likely reflects the fact that at very low doses, biological effects are driven by the complexity of individual ionization clusters within DNA-sized volumes. As the dose increases and, consequently, the number of particle tracks and interactions grows, macroscopic descriptors such as LET and microdosimetric quantities become increasingly relevant, as they capture cumulative effects and spatial correlations at larger scales.

The dependence between cell survival and radiation field description becomes more complex when the dose is considered, as illustrated in Figures 6, 7. Specifically, at lower doses, nanodosimetry plays a more prominent role. As the dose increases, the contribution from the other spatial scales becomes more significant. This trend is observed primarily at lower energies, consistent with earlier observations: low-energy carbon ions exhibit a strong dominance of nanodosimetry, with extremely low energies relying exclusively on this scale. This confirms the considerations made regarding the differences between *D*10 and the low-dose limit.

A further interesting point to discuss, as shown in Figures 4, 5, is the presence of a maximum in microdosimetric importance around 10 MeV/u (corresponding to LET ≈150 keV/μm), which is particularly evident for carbon ions. This LET value is precisely the threshold above which the overkill effect occurs, leading to a decrease in biological effectiveness. The observed attention pattern may be attributed to the complexity of describing biological effects in very high-LET scenarios, where the overkill phenomenon becomes significant for heavy ions. In such cases, the model requires a stronger contribution from microdosimetric descriptors to complement nanodosimetric information in predicting the biological outcome. This behavior supports the hypothesis that microdosimetric parameters act as sensitive indicators of radiation quality, especially in high-LET regimes. It is worth emphasizing that an accurate representation of the overkill effect has been achieved without relying on any saturated microdosimetric term, such as y^*^, which is commonly used in mathematical models to account for saturation. We deliberately avoided including y^*^ because this quantity incorporates not only physical aspects but also biological considerations. While y^*^ is widely adopted in the community to model the overkill effect, our data-driven approach naturally captures this phenomenon without introducing explicit correction terms.

Furthermore, Figure 5 provides additional insights into the specific contributions of each spatial scale. For protons, the impact of microdosimetry, particularly dominant at higher energies, is primarily captured by the fluence-averaged lineal energy ȳ*F*. As beam energy increases, the model assigns greater importance to ȳ*F*, while the relevance of dose-averaged lineal energy and nanodosimetric descriptors decreases. This suggests that the DL model naturally prioritizes ȳ*F* over LET, likely because microdosimetry offers a more accurate representation of large particle tracks produced by high-energy protons compared to the more aggregated LET metric. In such cases, fluence-averaged quantities provide a more representative characterization than dose-averaged metrics. In the low-dose limit, an interesting pattern emerges: the importance of $\bar{F}\left(1\right)$ increases with energy, whereas $\bar{F}\left(3\right)$ exhibits the opposite trend. This behavior is consistent with the physics of track structure; low-energy protons tend to produce larger ionization clusters, while higher-energy protons generate smaller clusters, making single-ionization probabilities more relevant at high energies. Furthermore, the contribution from microdosimetry is primarily driven by ȳ_*D*_, compared to *D*10 and the dose at 2 Gy, where the majority of the contribution is associated with ȳ_*F*_. This observation is consistent with the MKM, which postulates that the α parameter depends on radiation quality through ȳ_*D*_. Since the α parameter corresponds to the low-dose limit of the logarithmic survival curve, this provides the same information as the attention observed in the low-dose region.

Regarding carbon ions, generally, the main responsibility for nanodosimetry is $\bar{F}\left(1\right)$, as evident in Figure 5B, showing that a first-order quantity is already a good indicator of DNA damage clustering, with an enhanced strong contribution of $\bar{F}\left(3\right)$ at extremely lower energies that it is most likely used by the model to predict the biological effect at extremely low energies.

Beyond characterizing the radiation field itself, our results emphasize that variations in radiosensitivity across cell lines lead to distinct responses under the same irradiation conditions. Radiosensitivity is quantified through the ratio α_*X*_/β_*X*_ for a reference radiation, as commonly adopted in the community. This ratio, derived from the LQ model, is a key radiobiological parameter: tissues with a high α_*X*_/β_*X*_ value (e.g., most tumors) are dominated by the linear component and exhibit reduced sensitivity to fractionation, whereas tissues with a low α_*X*_/β_*X*_ value (e.g., late-responding normal tissues) are more sensitive to dose per fraction. This concept is fundamental in treatment planning, guiding fractionation strategies to balance tumor control and normal tissue sparing. While a threshold of approximately 10 Gy is typically considered, our dataset contains few experiments with α_*X*_/β_*X*_>10 Gy. Therefore, we adopt a threshold of 4 Gy for classification purposes. The analysis is restricted to protons because the dataset used for carbon ions is not uniformly tested across all energies, introducing bias. It is important to note that, in general, cell lines with higher α_*X*_/β_*X*_ ratios are less sensitive to variations in particle energy and, consequently, to differences in particle efficacy. This observation aligns with the current understanding that particles with higher LET are more robust against variations in cell-line radiosensitivity. Furthermore, attention at the nanodosimetric scale appears to be largely independent of the cell line, whereas the most significant differences for *D*10 and 2 Gy emerge at the microdosimetric level for higher-energy protons. In the low-dose limit, there is an increasing emphasis across the three scales–from LET to microdosimetry and nanodosimetry. Notably, LET plays a more critical role for cell lines with high α_*X*_/β_*X*_, whereas microdosimetry becomes more relevant for those with low α_*X*_/β_*X*_.

In general, this work has been inspired by the long-standing effort of the community to relate energy deposition to biological effect, as well as by recent results reported in Friedrich et al. (2018), where the authors pursued a similar goal through a model-based analysis. Our work shares the same overarching objective but adopts a purely data-driven perspective. Some analogies and differences emerge between the two approaches.

In particular, regarding contributions in the low-dose limit, Friedrich et al. (2018) reports a dominance of the micrometer scale for high-energy carbon ions and of the nanometer scale for low-energy carbon ions. We observe similar trends: nanodosimetry dominates for low-energy carbon ions, with reduced absolute attention at higher energies. However, even at high energies, nanodosimetry remains the most influential descriptor in our model.

Concerning the more macroscopic descriptor, which in Friedrich et al. (2018) corresponds to a measure around 10 μm, whereas in our case it is LET, we consistently report lower attention. Several possible explanations for this discrepancy exist, but we believe that the different characterization of the radiation field is the most relevant. Specifically, we employ microdosimetric and nanodosimetric descriptors, whereas (Friedrich et al., 2018) relies on the amorphous track model (Friedrich et al., 2013), which parameterizes radial energy deposition. This choice reflects the methodological foundations: the authors in Friedrich et al. (2018) build on the LEM model, while our data-driven approach offers greater flexibility for incorporating diverse predictors.

One of the key strengths of our approach lies in the use of the attention mechanism to provide native interpretability through its sequential, attention-based feature selection. Unlike *post-hoc* explainability methods such as SHAP, which often require building surrogate models and rely on permutation-based techniques, our approach provides transparent insights directly from the model itself. It is worth stressing that this does not mean that parameters within the model inherently carry physical meaning, but the role of each parameter is explicit. This is particularly advantageous in our context, where the correlation of different radiation quality metrics undermines the reliability of permutation-based explanations. In our model, feature correlations are addressed intrinsically through the sparsemax-based attention mechanism implemented in TabNet. This enforces sparsity in the attention maps, compelling the model to focus on a limited subset of features at each decision step, before a subsequent aggregation with different weights. As a result, highly correlated features are naturally down-weighted across the sequential decision process, improving both feature selection stability and model interpretability. This allows the model to capture diverse and complementary information while mitigating the effects of feature collinearity. At each of the three steps in the model developed in this research, a sparsemax activation function is applied to enforce selective attention to a limited number of variables. This mechanism selects features and mitigates issues related to feature correlation. Each step produces a prediction, which is subsequently recombined into the final output. This built-in transparency not only enhances trust in the model's predictions but also facilitates its potential translation into clinical settings, where interpretability is essential for decision support and regulatory acceptance.

One limitation of this work is that, although we extracted the beam type (monoenergetic or SOBP) from the PIDE database, this classification is inherently constrained. In practice, so-called monoenergetic beams are often produced through passive energy degradation, meaning they do not represent truly pristine beams. As a result, the distinction between monoenergetic and SOBP beams may be blurred, introducing ambiguity in beam characterization. Moreover, perfect beam characterization is not possible when relying on historical datasets such as PIDE, which often lack detailed metadata on beam delivery and modulation.

Another important consideration is the biological endpoint. Although the chosen endpoint represents the current gold standard, it is not necessarily the most relevant from a radiobiological perspective. Other endpoints, closer to *in vivo* conditions, could provide more clinically meaningful insights and should be integrated when data become available. Here we refer to tissue- or patient-level outcomes, such as NTCP-related measures or clinically observed toxicity endpoints, although their integration requires careful consideration of potential biases arising from heterogeneous clinical protocols and data collection procedures. The main drawback here is data availability; however, our approach is designed to be extensible and could incorporate additional endpoints if suitable datasets exist.

At last, it is worth mentioning that the investigation presented in this work relies on a set of radiation-field metrics that we believe are among the most relevant for describing the physical determinants of biological response. However, other descriptors could certainly be considered. Our choice was motivated by the limited size of the available dataset, which constrains the number of features that can be reliably included without introducing overfitting. To address this, we restricted the feature set while maintaining a balance between microdosimetric and nanodosimetric representations. In general, alternative selections are possible, and future studies with larger datasets could explore a broader range of physical descriptors to further enhance model performance and interpretability.

Finally, transparency is fundamental to ensure clinical translation. This is another strength of our approach: by maintaining interpretability and openness in the modeling process, we facilitate reproducibility and foster trust in the integration of AI into particle therapy.

Overall, our findings advocate for an integrative framework that unites physics-based descriptors at different spatial scales with biological cell characterization, ultimately advancing the precision and personalization of particle therapy treatments. To fully realize this potential, we also emphasize the need for more comprehensive and standardized reporting of radiobiological measurements. Improved documentation would enable more accurate Monte Carlo-based characterization of experimental conditions, particularly regarding beam properties and microenvironmental factors. Given our observation that multiple spatial scales significantly influence cell survival outcomes, it becomes increasingly clear that consistently capturing all relevant scales across experiments is challenging yet essential for robust, interpretable modeling.

## 5 Conclusions

This study demonstrates that attention-based DL can accurately predict cell survival and RBE in hadron therapy by integrating radiation descriptors across nanodosimetric, microdosimetric, and macroscopic scales. Attention analysis shows that no single scale dominates; instead, the model dynamically combines complementary information, with nanodosimetric and microdosimetric quantities being most influential and LET contributing modestly. The ion-specific attention patterns confirm the need for a multiscale approach to capture both microscopic and macroscopic effects. Our framework achieves high predictive accuracy (MAPE ≈ 10%) while integrating interpretability, highlighting AI as a valuable tool for radiobiological modeling and future clinical translation.

The present study highlights the potential of a data-driven approach to unravel the complex interplay between different scales of energy deposition, dose, and biological endpoints. These interactions reflect the underlying mechanisms of DNA damage induction, repair, and cell population dynamics that ultimately shape cell survival. Similar, if not more intricate, mechanisms can be expected for clinically relevant endpoints. Artificial intelligence offers a transformative opportunity to incorporate such detailed physical and biological descriptions into treatment-plan optimization. Importantly, this work demonstrates that AI is not only capable of achieving high predictive accuracy, as widely recognized, but can also provide transparency and interpretability, enabling us to understand the model's reasoning. This capability is fundamental for clinical adoption and trust in AI-driven decision support.

## Data Availability

Publicly available datasets were analyzed in this study. Data of the PIDE dataset available here: https://www.gsi.de/work/forschung/biophysik/forschungsfelder/radiobiological_modelling/pide_project.
